# Frequency and Localization of Second Primary Tumors in Patients with Oropharyngeal Carcinoma—The Influence of the Human Papilloma Virus

**DOI:** 10.3390/cancers13081755

**Published:** 2021-04-07

**Authors:** Salome L. Bosshart, Grégoire B. Morand, Martina A. Broglie

**Affiliations:** 1 Department of Otorhinolaryngology, Head and Neck Surgery, University Hospital Zurich, Frauenklinikstrasse 24, 8091 Zurich, Switzerland; salome.bosshart@uzh.ch (S.L.B.); gregoire.morand@mail.mcgill.ca (G.B.M.); 2 Faculty of Medicine, University of Zurich, 8032 Zurich, Switzerland

**Keywords:** human papillomavirus, second primary, oropharyngeal squamous cell

## Abstract

**Simple Summary:**

Human papillomavirus (HPV) infection, smoking, and excessive alcohol consumption have been established as risk factors for the development of oropharyngeal squamous cell carcinoma (OPSCC). While the HPV epidemic has led to an increasing incidence of OPSCC, HPV-negative OPSCC cases associated with smoking and alcohol remain stable. As HPV-positive and -negative OPSCC present two distinct etiological, clinical, and prognostic entities, different treatment and follow-up strategies are being discussed. Still, specific surveillance strategies for HPV-positive OPSCC are lacking, as the risk of second primary tumors (SPT) in the era of HPV-associated OPSCC has not been comprehensively assessed. Our study investigated the frequency and localization of SPT of HPV-positive OPSCC, as well as their prognostic impact. We find that the SPT of HPV-positive OPSCC are less frequent than those of HPV-negative OPSCC, and they are also associated with higher survival rates. The localization of SPT of HPV-positive OPSCC did not differ from the localization of SPT of HPV-negative OPSCC.

**Abstract:**

Purpose: To investigate the frequency, localization, and survival of second primary tumors (SPT) of oropharyngeal squamous cell carcinoma (OPSCC) depending on human papillomavirus (HPV) status. Methods: We performed a retrospective chart analysis of 107 OPSCC patients treated at the Zurich University Hospital from 2001 to 2010. Rate and localization of SPT after an index OPSCC were stratified according to smoking and HPV infection status. Results: In total, 57/91 (63%) included patients showed an HPV-associated OPSCC. Of these, 37/57 (64.9%) patients with an HPV-positive and 32/34 (94.1%) patients with an HPV-negative OPSCC were smokers. The median age at diagnosis of the SPT was 59.54 years (interquartile range 52.7–65.6). In addition, 8/57 (14%) HPV-positive and 13/34 (38.2%) HPV-negative patients developed SPT. The rate of SPT in patients with HPV-positive index tumors was significantly lower than in patients with HPV-negative OPSCC (*p*-value 0.01). Smokers showed significantly more SPT in the head and neck area than outside. The development of an SPT led to a significantly lower survival time in HPV-negative patients, while it did not significantly affect the survival time of HPV-positive patients. Conclusions: Patients with HPV-positive index tumors had a significantly lower risk of developing SPT than patients with HPV-negative tumors. If SPT developed, survival was significantly shorter in patients with HPV-negative tumors than with HPV-positive tumors.

## 1. Introduction

Oropharyngeal squamous cell carcinomas (OPSCC) are rare. Major risk factors for OPSCC include excessive tobacco and alcohol consumption [1]. In 1946, Slaughter [2] presented the model of field carcinogenesis, according to which the mucosa of the upper aerodigestive tract has an increased risk of oncogenic degeneration due to continuous exposure to noxious agents. Based on this observation, panendoscopy (laryngopharyngoscopy, bronchoscopy, and esophagoscopy) has been established for the workup of patients with newly diagnosed HNSCC to detect synchronous second primary tumors (SPT) [3]. SPT are observed in approximately 10–20% of patients with exposure to smoking or excessive alcohol consumption [4,5].

High-risk human papillomavirus (HR-HPV) infections have been known to cause genital tumors for about 3 decades [6]. About 2 decades ago, HPV infections were also recognized as a risk factor for OPSCC [1]. In developed countries, the incidence of all HNSCC has decreased—possibly due to declining tobacco exposure [7]. Meanwhile, the incidence of OPSCC has increased over the past three to four decades [8]. This specific incidence increase can be attributed to the increasing prevalence of oncogenic HPV infections in the oropharynx [9,10]. Thus, HPV has been identified as a new risk factor specific for OPSCC that shows little impact on other HNSCC [11]. HPV-associated OPSCC increasingly occur in individuals without the usual exposure to alcohol and tobacco [12].

HPV-induced OPSCC present an etiologically, molecularly, clinically, and prognostically distinct entity from tobacco- and alcohol-associated OPSCC [13,14,15,16]. SPT seem to occur less frequently in patients with HPV-positive OPSCC than in patients with HPV-negative OPSCC [17]. Therefore, a modulation of the follow-up strategy for HPV-positive OPSCC has to be considered.

The aim of this study was to analyze the frequency and localization as well as the prognostic significance of SPT after index OPSCC in relation to the risk factors HPV infection and smoking. Our research questions were the following: (i) How common are SPT in a consecutive cohort of patients with OPSCC? (ii) Are SPT more common in smokers than in nonsmokers? (iii) Does this frequency depend on HPV status? (iv) Does the location of SPT differ between smokers and nonsmokers or HPV-positive and HPV-negative patients? (v) What is the impact of the development of SPT on survival?

## 2. Materials and Methods

A retrospective analysis was performed using medical records of patients who were treated in a curative intent for newly diagnosed oropharyngeal carcinoma (OPSCC) at the Department of Otorhinolaryngology—Head and Neck Surgery at the University Hospital Zurich (USZ), Zurich, Switzerland between 2001 and 2010.

Inclusion criteria were an index tumor in the oropharynx. Exclusion criteria on the other hand were a non-evaluated HPV status. The starting point of observation was the diagnosis of the index tumor. Endpoint of observation was either last follow-up examination until January 2019 or death.

### 2.1. Analysis of Index Tumors

HPV positivity was determined according to an accepted algorithm [18], which includes p16 immunohistochemistry and PCR detection of HPV DNA (high-risk types). P16-positive and HPV DNA-negative tumors were considered HPV-negative.

Furthermore, age at diagnosis of the index tumor, gender, location of the index tumor, tobacco and alcohol consumption, occurrence of SPT, and disease progression (recurrence, death of patients) were obtained from medical records. Death was distinguished as due to index tumor, due to SPT, and independent of cancer. Survival rates were plotted using Kaplan–Meier survival curves, with the start of observation being the time of diagnosis of the index tumor.

- Smokers were defined as ≥10 pack years, regardless of the time of tobacco use.
- Alcohol abuse was defined as a history of >2 U/day for women and >3 U/day for men, respectively, with a standard unit (U) corresponding to 10 g of pure alcohol.
- An SPT was defined as a second carcinoma in the upper aerodigestive tract, lung, or bladder, differing in site from the index tumor, which was unlikely to be a metastasis of the index carcinoma [19].
- HPV types 16, 18, 33, 35, 56, and 59 were considered high-risk HPV types (HR-HPV) [20].

Age, sex, history of alcohol abuse, recurrence of index tumor, death from OPSCC-associated cancer, and development of second carcinomas were compared among the following three groups of patients: (1) HPV-positive nonsmokers, (2) HPV-positive smokers, and (3) HPV-negative patients.

### 2.2. Analysis of Second Tumors

Time to SPT occurrence was defined as time between diagnosis of index OPSCC and initial diagnosis of SPT.

Frequency and location of SPT after an index OPSCC were determined in relation to smoking and HPV status. Among SPT, synchronous tumors (detected simultaneously or within 6 months of the index tumor diagnosis) were distinguished from metachronous tumors (detected >6 months after the index tumor) [21]. In addition, the development of SPT of HPV-positive and HPV-negative patients was plotted over time.

### 2.3. Statistics

Statistical analyses of the data were performed using Stata 13. Patient characteristics were evaluated using T-test and Fisher’s exact test of group means controlled for unequal variances of individual characteristics. For evaluation of statistical significance of continuous variables such as age, the T-test was applied, while for discrete variables, Fisher’s exact test was applied. Statistical significance was defined at the 5% level of significance, i.e., *p* < 0.05.

To evaluate the effect of HPV status, UICC stage (Union internationale contre le cancer), and therapy mode, age, sex, tobacco, and alcohol abuse on the development of SPT, HPV status, and death from cancer, multivariate Cox regression analyses were performed.

For survival analysis, we used Kaplan–Meier survival curves and controlled for significant differences between groups using log-rank tests. Additionally, a multivariate cox regression analysis was performed to evaluate the influence of age, gender, tobacco and alcohol abuse, HPV status, index-UICC stage, therapy mode of the index tumor, and development of an SPT on survival.

### 2.4. Ethics

Ethical approval by the Zurich Ethics Committee for retrospective analysis of patient data is available (KEK-ZH-No. 2013-0019).

## 3. Results

### 3.1. Patient Characteristics

After exclusion of 16 patients due to undefined HPV status, the analyzed cohort consisted of 91 patients. Table 1 summarizes the patient characteristics. Follow-up of the initial carcinoma occurred for a mean of 57.17 months (4.76 years, interquartile range 31.08–65.25 years) from diagnosis.

In total, 57/91 OPSCC (62.6%) showed p16-overexpression and positivity for HR-HPV DNA, as detected by PCR. Therefore, these tumors were considered as HPV-positive tumors. With 89.5% (51/57 HPV-positive tumors), HPV16 was the most frequently identified HPV type. Other HPV types detected were HPV18, HPV 33, HPV 35, HPV 56, and HPV 59.

More than 90% of the index tumors of HPV-positive patients were located in the tonsils, as shown in Table 1. The index tumors of HPV-negative patients were located in the tonsil (76.5%), base of tongue (14.7%), pharyngeal wall (5.9%), and soft palate (2.9%).

The mean age of all patients with OPSCC was 59.54 years and comparable among the three groups (HPV-positive smokers, HPV-positive nonsmokers, and HPV-negative smokers).

Independent of HPV status, OPSCC patients were more likely to be male (74.7%) and smokers (72.9%). HPV-positive patients smoked significantly less than HPV-negative patients (*p*-value 0.002). Only 2/34 patients with HPV-negative tumors were nonsmokers and therefore, they were not separately analyzed.

There were no patients with history of alcohol abuse among nonsmokers, whereas 9/37 (24.3%) of the HPV-positive smokers and 19/32 (59.3%) of the HPV-negative smokers had a history of alcohol abuse. This difference in alcohol consumption between smokers and nonsmokers was statistically highly significant (*p*-value 0.0001).

### 3.2. Frequency of Second Primary Tumors

In the overall cohort, 21/91 (23.1%) OPSCC patients developed an SPT during the observation period (Table 2): 4 (20%) HPV-positive nonsmokers, 4 (10.8%) HPV-positive smokers, and 13 (38.2%) HPV-negative patients. HPV-negative OPSCC patients developed significantly more SPT than HPV-positive patients (*p*-value 0.011, Fisher’s exact test), whereas there was no difference between HPV-positive nonsmokers and HPV-positive smokers.

Using a multivariate Cox regression analysis, we examined the influence of HPV-positivity, smoking, alcohol, age, gender, UICC stage, and the therapy mode for the index tumor, on the development of SPT. The results of the multivariate Cox regression are shown in Table 3. Factors such as smoking status, alcohol abuse, age, and gender of the patients showed no significant influence on the development of SPT in this analysis. HPV positivity, on the other hand, was with an HR of 0.344 (95% CI 0.149–0.791, *p* = 0.012), which was statistically significantly inversely correlated with the development of SPT.

### 3.3. Distribution of Second Primary Tumors over Time

Six of 21 (28.6%) SPT appeared synchronously with the index tumor, while 15/21 (71.4%) appeared metachronously. The synchronous SPT were located in the oropharynx (2 SPT), hypopharynx (2 SPT), larynx (1 SPT), and lung (1 SPT).

Metachronous SPT appeared after an average of 68.9 months (range: 11–190 months, IQR 20–101 months). The majority (60%) of the metachronous SPT was located inside the head and neck area (Table 4): oral cavity (4 SPT), oropharynx (3 SPT), and larynx (2 SPT). 

One case showed a metachronous SPT in the esophagus, and 5 were located in the lung.

**Figure 1:**
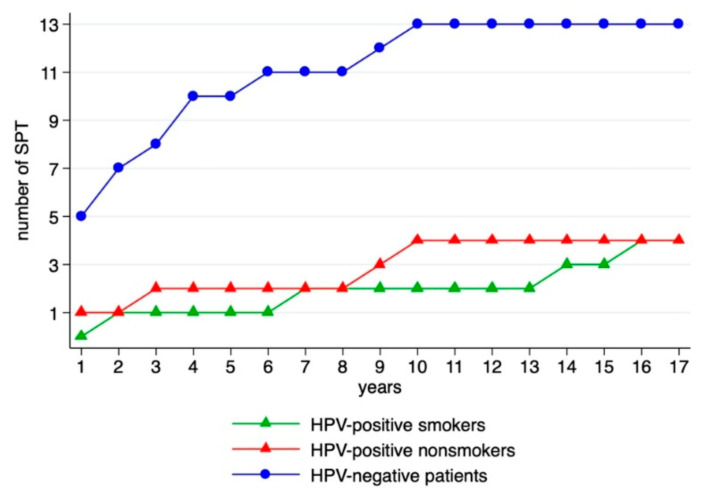
Distribution of SPT over time.

The number of SPT and timely distribution of SPT occurrence in relation to the HPV status are depicted in Figure 1.

Metachronous SPT of HPV-positive patients occurred after an average of 91.6 months, while the SPT of HPV-negative patients were diagnosed after 49 months on average. Although this difference is striking, it did not reach statistical significance (*p*-value of 0.161, Table 5).

Notably, two of the HPV-positive SPT were diagnosed after more than 12 and 15 years, respectively (larynx and oropharynx).

### 3.4. Influence of Second Primary Tumors on Survival

**Figure 2:**
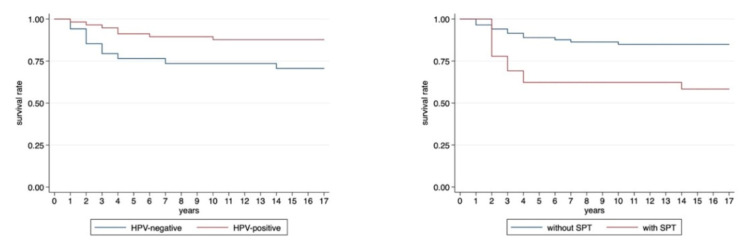
Kaplan–Meier survival plots of HPV-positive and HPV-negative patients. (Log-rank test: *p*-value = 0.037) and of patients with and without SPT (Log-rank test: *p*-value = 0.02).

Overall, 17/91 (18.7%) OPSCC patients died: 2 HPV-positive nonsmokers, 5 HPV-positive smokers, and 10 HPV-negative patients. Patients with HPV-positive OPSCC showed significantly better disease-specific survival than patients with HPV-negative OPSCC (5 year survival 91.2% for HPV-positive vs. 76.5% for HPV-negative patients; *p* < 0.0001, Figure 2), as shown by Kaplan–Meier analysis.

Five out of 21 (23.8%) OPSCC patients with SPT and 12/70 (17.1%) patients without SPT died. OPSCC patients without SPT had significantly better disease-specific survival than OPSCC patients with SPT (5-year survival 88.5% without SPT vs. 69.2% with SPT; Fisher’s exact test: *p*-value < 0.0001, Figure 2).

**Figure 3:**
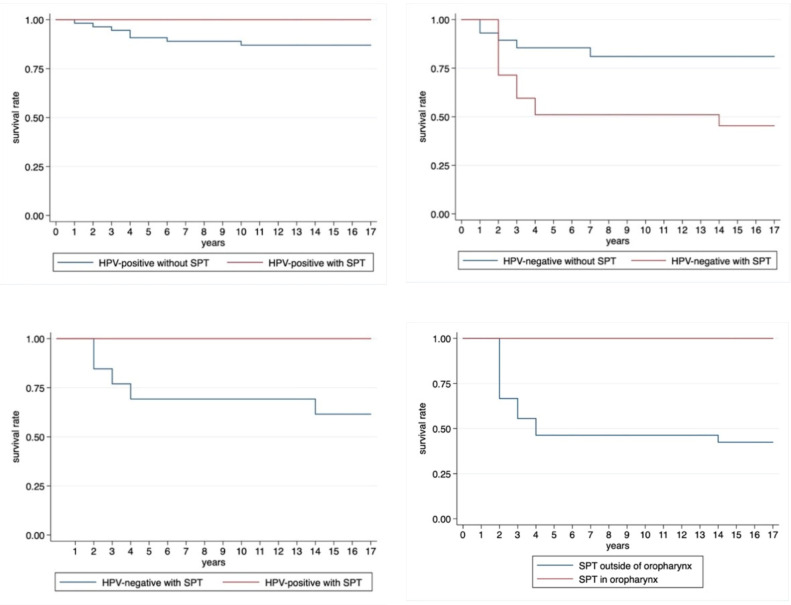
Kaplan–Meier survival plots of HPV-positive patients with and without SPT. (Log-rank test: *p*-value = 0.514), Kaplan–Meier survival plot of HPV-negative patients with and without SPT (Log-rank test: *p*-value = 0.033), of HPV-negative and HPV-positive patients with SPT (Log-rank test: *p*-value = 0.054), and of patients with SPT in and outside the oropharynx (Log-rank test: *p*-value = 0.054).

Out of 57 HPV-positive OPSCC patients, seven (12.3%) died from the index tumor and eight (14%) developed an SPT from which no one died within the observation period. There was no significant difference in survival in the HPV-positive patient group with SPT compared to HPV-positive patients without (Log-rank test: *p*-value = 0.514, Figure 3).

Out of 34 HPV-negative patients, 5/21 (23.8%) died without SPT and 5/13 (38.5%) died with SPT. The disease-specific 5-year survival rate was statistically significantly higher in HPV-negative patients without SPT (83.3%) than in HPV-negative patients with SPT (51.4%, Fisher’s exact test: *p*-value < 0.0001).

Figure 3 also shows the Kaplan–Meier survival plot of the 21 patients with SPT, analyzed by the HPV positivity of the index tumor. There was a trend for a worse survival in HPV-negative patients developing SPT compared to HPV-positive patients with SPT (log-rank test: *p*-value = 0.054).

Interestingly, none of the patients with a SPT in the oropharynx died during the time of observation. This stands in contrast to 23.8% deaths among patients with an SPT outside the oropharynx. However, the difference was not statistically significant (Kaplan–Meier survival plot in Figure 3, log-rank test: *p*-value 0.105).

As shown in Table 6, neither therapy mode nor UICC stage of the index tumor showed significant influence on OPSCC-associated survival.

## 4. Discussion

SPT of HPV-associated OPSCC have been investigated in search of optimal diagnostic workup strategies [22,23]. As shown by Hsu et al. [24], SPT present a major obstacle improving the long-term survival of patients with OPSCC. Therefore, SPT are of imminent importance to follow-up of OPSCC patients. Still, to our knowledge, this is the first study investigating the long-term survival of HPV-positive OPSCC patients with and without SPT.

Our study compares the frequency, localization, and timely distribution of SPT development in HPV-positive and HPV-negative OPSCC patients. Additionally, a comprehensive analysis of the influence of risk factors on SPT development and SPT localization is given. We report a significantly lower rate of SPT in HPV-positive OPSCC compared to HPV-negative OPSCC. Our survival analysis showed a significantly negative impact of SPT on survival in HPV-negative patients, while it did not significantly affect the survival time of HPV-positive patients. Due to the small number of SPT, especially in the cohort of HPV-positive patients, no firm conclusions on SPT localization can be made.

The focus of the latest research on HPV-associated OPSCC has been on possible therapy modifications [25]. With the presented results, we hope to extend the discussion to follow-up protocol for HPV-positive OPSCC patients.

### 4.1. Patient Characteristics

The three main risk factors for OPSCC are smoking, alcohol abuse, and HPV infection [26]. Indicating the impact of HPV infections, our cohort showed 63% HPV-associated OPSCC. Older literature had found HPV-associated OPSCC to be present in a group of patients who are younger than HPV-negative OPSCC patients and have less exposure to noxious agents. [12]. However, more recent results from the US show a “moderation of increasing incidence in younger individuals and a shift in the burden to older individuals” [27]. Consistent with the increasing number of HR-HPV infections in the oropharynx [8], HPV-positive nonsmokers (all without alcohol abuse) accounted for a remarkable 22% of our cohort. In addition, HPV-positive patients smoked significantly less than HPV-negative patients. The fraction of smokers among HPV-negative OPSCC patients was high at 94.1%. Excessive alcohol consumption was only found in smokers and was more prevalent in HPV-negative patients. The association of heavy tobacco and alcohol abuse is well known in the literature [28].

Contrary to other studies [13,29], in our study cohort, HPV-positive patients were not younger than HPV-negative patients.

### 4.2. Localization of Index Tumors

Consistent with literature [30,31], the vast majority of HPV-positive index OPSCC in our cohort was located in the tonsils (93%) and the base of the tongue (7%). This supports the suggestion of the predilection of HPV-associated tumors in the lymphoid tissue of Waldeyer’s ring [32,33,34].

In contrast, HPV-negative patients showed a broader distribution with index tumors in tonsil (76.6%), base of tongue (14.7%), pharyngeal wall (5.9%), and soft palate (2.9%).

### 4.3. Frequency and Localization of Second Primary Tumors

In this study, the definition of SPT was based on Warren and Gates [19], even though the latest and more exact definition of SPT goes back to Braakhuis et al. [35]. Due to the retrospective nature of this study, data on genetic patterns of the SPT were not available. Therefore, the genetic differentiation of SPT from recurrence and metastasis, as suggested by Braakhuis et al. [35], was not applicable. Table 7 provides an overview over the localization and histology of all SPT in our cohort.

As hypothesized, HPV-positive patients developed significantly fewer SPT (15.79%) than HPV-negative patients (38.24%) [17,21,36]. Similarly, Martel et al. [15] performed a study with 412 OPSCC patients, including T and N category as additional risk factors, and they found only HPV status showing a significant impact on the occurrence of SPT. The authors found a 4.5-fold increased risk of developing SPT in HPV-positive patients with heavy abuse of noxious agents or HPV-negative patients with moderate or no abuse of noxious agents, compared with HPV-positive patients without exposure to noxious agents. The risk of HPV-negative patients with severe noxious exposure was even increased by a factor of 13.2 compared with HPV-positive patients without exposure to extrinsic carcinogens. This illustrates the lower SPT risk of HPV-positive OPSCC, even with the influence of tobacco/alcohol abuse.

Contrary to expectations, we did not find a significantly increased incidence of SPT in smokers compared to nonsmokers, even with statistical control for age, sex, HPV status, and alcohol abuse (Table 3). Putting this result into perspective, Table 8 showed a significant influence of smoking on the occurrence of SPT in the head and neck area. We attributed the nonsignificant influence of smoking on SPT development in Table 3 to the small number of cases in our cohort. Milliet et al. [36] found alcohol and tobacco consumption, as well as a negative HPV status to be significant predictors for SPT development in OPSCC patients.

In a literature review of the last 40 years (1979–2019), Coca-Pelaz et al. [5] found that SPT after HNSCC occurred most frequently in the head and neck region, followed by lung and esophagus. In our cohort, the head and neck area and the lung were also the most common location of SPT. The same distribution was shown for HPV-positive patients, independent of tobacco use as a risk factor. Exemplarily, an HPV-positive nonsmoker of our cohort developed a SPT in the oral cavity. This stands in contrast to the hypothesis of Rietbergen et al. [37] saying that SPT of HPV-positive OPSCC do not occur in the head and neck region due to absent field carcinogenesis. They examined the tumor-free resection margins of HPV-positive OPSCC and were unable to detect HPV16-E6 mRNA [37]. From this, they concluded that field carcinogenesis by HPV is unlikely. McGovern et al. [38] examined the mucosa between two adjacent HPV-positive OPSCC and were not able to detect HPV, either.

In our study, HPV-positive patients mostly showed metachronous SPT. This in agreement with the results of Xu et al. [23], who showed that the diagnostic workup of HPV-positive OPSCC detected less synchronous SPT than in HPV-negative OPSCC. Thus, field cancerization by HPV, as first described by Slaughter in 1946 [2], seems unlikely for HPV-associated tumors.

On the basis of the clonal relationship of first and second tumors [39], Rietbergen et al. [37] postulated that synchronous SPT of HPV-positive OPSCC result from the same infection as the index tumor. They also deduce that SPT arise from the migration of HPV-infected cells. A similar phenomenon has been described in women with cervical carcinoma and SPT in the lower genital tract (vulva and vagina) [40].

In a case report of a 46-year-old patient with three synchronous HPV-associated OPSCC and two cervical metastases, McGovern et al. [38] also commented on the phenomenon of multiple HPV-associated tumors. After excluding field carcinogenesis by HPV, McGovern et al. postulated two models for the development of second HPV-associated lesions: (1) multiple independent HPV infections leading to the development of multiple tumors, and (2) a primary HPV-associated tumor producing clonally related neoplasms that migrate to new localizations and appear as metachronous SPT.

The latter theory was supported by the findings of a case report of Joseph et al. [39], who showed four HPV-associated carcinomas of the tonsils with molecularly identical SPTs in the respective contralateral tonsil. In view of these data, the question arises as to the exact delineation between SPT and metastasis. A 100% genetic match would classify for a metastasis, even if it appeared with an exceptionally large temporal distance from the primary tumor.

Due to the small number of HPV-positive SPT in our cohort, we could not make robust statements on the frequency of HPV-associated SPT at individual sites. Still, the detected localizations, especially of HPV-positive SPT, are noteworthy.

In our cohort, looking specifically at SPT in the head and neck area, tumoral HPV positivity of the index tumor did not show any significant influence on the frequency of SPT, while smoking and age of the patient did. Thus, the risk of SPT in the head and neck area decreased with increasing age, while it increased in smokers.

This is consistent with the results of Peck et al. [22] who demonstrated that HPV-seropositive patients developed proportionally fewer SPT in traditionally tobacco-associated sites than HPV-seronegative patients. However, it is important to note that Peck et al. [22] determined HPV status using serum markers, which show exceptionally high specificity [41,42] but may precede tumor development by years [43].

### 4.4. Distribution of Second Primary Tumors over Time

Tumor follow-up was conducted over an average of 4.76 years after diagnosis, which is in line with other studies on the same topic [5]. Still, our cohort contained patients that were observed over 17 years.

As Kreimer et al. [43,44] found in serologic studies, more than 10 years can elapse between HPV infection and the appearance of oropharyngeal cancer.

From Figure 1, we find that the majority of SPT occurred metachronously; in particular, SPT of HPV-positive OPSCC. HPV-positive metachronous tumors were detected an average of 42.13 months later than HPV-negative ones, and two of the SPT did not appear until more than 13 years after diagnosis of the index tumor. Thus, it seems important to maintain follow-up over time, especially for HPV-positive OPSCC. To determine SPT risk at different points in time after diagnosis of the index OPSCC, studies with long-term follow-up of larger cohorts than ours are needed.

### 4.5. Prognostic Impact of SPT

The Kaplan–Meier plot in Figure 2 shows a significantly higher survival rate of HPV-positive patients compared to HPV-negative patients. This is consistent with the results of many studies and with the landmark paper by Ang et al. [45]. They showed a more than 50% lower mortality risk in HPV-positive versus HPV-negative OPSCC patients.

Looking at the impact of SPT on the prognosis of OPSCC patients, the consensus in the literature is the following: despite advances in therapy, the survival rate of OPSCC patients remains reduced, especially if SPT are developed [24,46]. Bhattacharyya and Nayak [47] found an up to 50% decreased survival rate in HNSCC patients with SPT compared to HNSCC patients without SPT. Milliet et al. [36], on the other hand, did not find a difference in overall survival between patients with and without SPT. In line with the results of Bhattacharyya and Nayak [47], our cohort also showed significantly worse survival of patients with SPT compared to patients without SPT. An analogous result was observed when looking at HPV-negative patients only, who showed significantly worse survival rates when SPT occurred (Figure 3). These findings were confirmed by the results of Martel et al. [15].

In contrast, in HPV-positive OPSCC patients, survival with SPT did not significantly differ from survival without SPT (Figure 3). The fact that survival in HPV-positive patients does not significantly worsen with the occurrence of SPT may be due to the better treatment response of HPV-positive tumors, as it has been shown in HPV-positive index tumors [48,49]. To our knowledge, no comparable study of survival with and without SPT has been published so far.

### 4.6. Strenghts and Limitations of the Study

This study is limited by the small number of cases for analysis of SPT. Since SPT are much less frequent in HPV-positive OPSCC than in HPV-negative OPSCC, we only recorded eight SPT of HPV-positive OPSCC. Thus, conclusions about the frequencies of specific SPT localizations are limited, but they provide a lead for larger prospective studies.

Retrospective analyses, as performed in this study, must be interpreted with caution due to the limited quality of retrospectively collected data, sample size limitations, and the potential for noncontrolled influencing factors.

Although this study focused on SPT of HPV-associated OPSCC, HPV status of the SPT was concluded solely based on the location and the HPV status of the index OPSCC. Therefore, uncertainty remains regarding the HPV status of SPT. The possibility remains, especially with tumors of the lung, that SPT are actually single metastases. Absolute certainty in this regard can only be obtained by genetic sequence analysis, which was not investigated in this study.

A strength of our study is the long observation period of up to 17 years.

## 5. Conclusions

HPV-positive OPSCC showed a lower risk of SPT than HPV-negative OPSCC. SPT of HPV-negative OPSCC occurred in the typical tobacco- and alcohol-associated sites. Significantly more HPV-negative patients died from index OPSCC or its SPT than HPV-positive patients. Thus, HPV positivity is a positive prognostic factor not only in the context of overcoming cancer but also in terms of long-term survival. Since SPT of HPV-positive OPSCC still occurred after more than 10 years, follow-up should not be reduced despite the lower SPT risk of HPV-positive OPSCC.

## Figures and Tables

**Table 1:** Patient characteristics. The *p*-values related to age are calculated by T-test (columns 5 and 6, marked in italics). The other group comparisons of columns 5 and 6 were performed with two-sided Fisher’s exact test. Values statistically significant at the 5% significance level are marked with *. Abbreviations: − = negative, + = positive, chemo = chemotherapy, IQR = interquartile range, RT = radiotherapy.

Patient Characteristics		1	2	3	4	5	6
		Total	HPV+ Nonsmokers	HPV+ Smokers	HPV− Patients	*p*-Values HPV+ vs. HPV− (*n* = 57 vs. *n* = 34)	*p*-Values Smokers vs. Nonsmokers (*n* = 69 vs. *n* = 22)
		*n* = 91	*n* = 20	*n* = 37	*n* = 34		
Age at diagnosis of index tumor	Median (y)	59.54	58.2	58.7	57.6	*0.483*	*0.646*
	IQR	52.7–65.6	52.8–66.7	51.0–67.3	53.8–65.1		
Sex							
male	*n* (%)	68 (74.7%)	14 (70%)	28 (75.7%)	26 (76.5%)	0.809	0.413
female	*n* (%)	23 (25.3%)	6 (30%)	9 (24.3%)	8 (23.4%)		
History of alcohol abuse	*n* (%)	28 (30.8%)	0	9 (24.3%)	19 (55.9%)	0.000 *	0.000 *
Index-OPSCC localization							
						
Tonsils	*n* (%)	79 (86.8%)	19 (95%)	34 (91.9%)	26 (76.6%)	0.050 *	0.281
Base of tongue	*n* (%)	9 (9.9%)	1 (5%)	3 (8.1%)	5 (14.7%)	0.286	0.446
Soft palate	*n* (%)	1 (1.1%)	-	-	1 (2.9%)	-	-
Pharyngeal wall	*n* (%)	2 (2.2%)	-	-	2 (5.9%)	-	-
Death due to OPSCC or associated cancer	*n* (%)	17 (18.7%)	2 (10%)	5 (13.4%)	10 (29.4%)	0.054	0.226
Index-UICC stadium							
I	*n* (%)	15 (16.5%)	3	5	7	0.56	1
II	*n* (%)	17 (18.7%)	5	6	6	1	0.345
III	*n* (%)	11 (12.1%)	0	4	7	0.093	0.06
IVa	*n* (%)	47 (51.6%)	12	22	13	0.055	0.47
IVb	*n* (%)	1 (1.1%)	0	0	1	-	-
Treatment							
Surgery	*n* (%)	91 (100%)	20 (100%)	37 (100%)	34 (100%)	1	1
RT ± chemo	*n* (%)	60 (65.9%)	13 (65%)	25 (67.6%)	21 (61.8%)		

**Table 2:** Overview of the number and locations of SPT of HPV-positive nonsmokers, HPV-positive smokers, and HPV-negative patients. The table compares the number of SPT, as well as the number of SPT at specific locations, between HPV-positive and HPV-negative patients, smokers and nonsmokers, and between HPV-positive nonsmokers and HPV-positive smokers using a two-sided Fisher’s exact test. Results statistically significant at the 5% significance level are marked with *. Abbreviations: HPV = human papillomavirus, NS = nonsmokers, S = smokers, + = positive, − = negative, SPT = second primary tumors.

Number and Localization of SPT	1	2	3	4	5	6	7
	Total Cohort *n* = 91	HPV+ NS *n* = 20	HPV+ S *n* = 37	HPV− *n* = 34	*p*-Values HPV+ vs HPV− (*n* = 57 vs. *n* = 34)	*p*-Values S vs. NS (*n* = 69 vs. *n* = 22)	*p*-Values HPV+ NS vs. HPV+ S (*n* = 20 vs. *n* = 37)
SPT							
Yes	21 (23.1%)	4 (20%)	4 (10.8%)	13 (38.2%)	0.011 ^*^	0.772	0.432
SPT localization							
Oral cavity	4 (4.4%)	1 (5%)	0	3 (8.8%)	-	-	-
Oropharynx	5 (5.5%)	0	2 (5.4%)	3 (8.8%)	0.965	0.020 *	0.429
Larynx	3 (3.3%)	0	1 (2.7%)	2 (5.9%)	-	-	-
Hypopharynx	2 (2.2%)	0	0	2 (5.9%)	-	-	-
Esophagus	1 (1.1%)	0	0	1 (2.9%)	-	-	-
Lung	6 (6.6%)	3 (15%)	1 (2.7%)	2 (5.9%)	0.146	0.053	0.486

**Table 3:** Multivariate Cox regression for SPT development. Table 3 shows the results of the multivariate Cox regression of SPT (0 = no SPT, 1 = SPT) on the independent variables of tumor HPV positivity (negative = 0, positive = 1), smoking status (0 = <10 py, 1 = ≥10 py), history of alcohol abuse (0 = no alcohol abuse, 1 = alcohol abuse), age (in years), sex (0 = male, 1 = female), UICC stage (1 = stage I, 2 = stage II, 3 = stage III, 4 = stage IVa, 5 = stage IVb), and therapy mode (0 = surgery only, 1 = RT ± chemo). * indicates statistical significance at the 5% significance level. Since alcohol use is unknown in three patients, 88 observations are included instead of 91. Abbreviations: Chemo = chemotherapy, CI = confidence interval, HR = hazard ratio, py = pack years, RT =radiotherapy, UICC = Union internationale contre le cancer.

Multivariate Cox Regression for SPT Development	SPT Development	
Parameter	HR (95% CI)	*p*-Value
Tumor HPV-positivity	0.362 (0.155–0.847)	0.019 ^*^
Smoking (>10 py)	0.723 (0.239–2.190)	0.566
Alcohol abuse	0.995 (0.456–2.170)	0.989
Age	0.981 (0.940–1.023)	0.365
Gender	0.370 (0.092–1.488)	0.161
UICC stage	0.908 (0.659–1.251)	0.556
Therapy mode	0.911 (0.403–2.058)	0.823
Number of observations	88	

**Table 4:** Number of SPT in and outside the head and neck area in HPV-positive and HPV-negative patients. The *p*-value compares HPV-positive and HPV-negative patients regarding SPT localization in the head and neck area. The *p*-value was calculated using Fisher’s exact test.

SPT Localizations	Total *n* = 21 (%)	HPV-Positive (*n* = 8)	HPV-Negative (*n* = 13)	*p*-Value
Head and neck	14 (66.7%)	4 (50%)	10 (76.9%)	0.346
outside head and neck	7 (33.3%)	4 (50%)	3 (23.1%)	

**Table 5:** Time of occurrence of metachronous SPT.

Time of Occurrence of Metachronous SPT (Months)	HPV-Positive (*n* = 7 SPT)	HPV-Negative (*n* = 8 SPT)	*p*-Value
Average	91.6	49	
Median	90	39.5	0.161
IQR	22–154	17–77	

**Table 6:** Multivariate Cox regression for death in association with OPSCC. Table 6 shows the influence of the following variables on death due to OPSCC and/or SPT after OPSCC: tumor HPV positivity (negative = 0, positive = 1), smoking status (0 = <10 py, 1 = ≥10 py), alcohol status (0 = no alcohol abuse, 1 = alcohol abuse), age (in years), SPT development (0= no SPT, 1 = SPT), therapy mode (1 = surgery, 2 = RT, 3 = surgery + RT, 4 = surgery + RT + chemotherapy, 5 = surgery + chemotherapy), and UICC stage of the index OPSCC (1 = stage I, 2 = stage II, 3 = stage III, 4 = stage IVa, 5 = stage IVb). Abbreviations: CI = confidence interval, HR = hazard ratio, py = pack years, RT = radiotherapy.

Multivariate Cox Regression for Death Associated with OPSCC	Death Associated with OPSCC	
Parameter	HR (95% CI)	*p*-Value
Tumor HPV-positivity	0.519 0.733–1.459)	0.195
Smoking (>10 py)	1.634 (0.372–7.182)	0.515
Alcohol abuse	1.248 (0.462–3.373)	0.662
Age	1.024 (0.981–1.069)	0.276
SPT development	0.978 (0.404–2.364)	0.960
Treatment of index tumor	1.034 (0.733–1.459)	0.850
Index UICC stage	0.871 (0.580–1.308)	0.505
Number of observations	88	

**Table 7:** Overview over localization and histological entity of the tumors of HPV-positive and HPV-negative patients who developed an SPT.

HPV Positivity	Localization of Index Tumor	Localization SPT	Histology of SPT
Positive	tonsil	lung	Squamous cell carcinoma, with growth pattern distinct from index tumor
	Tonsil	Lung	Squamous cell carcinoma
	Base of tongue (right)	Vallecula (left)	Squamous cell carcinoma
	Base of tongue	Lung	Small cell lung carcinoma
	Oropharynx	Lung	Squamous cell carcinoma
	Tonsil	Vocal cord	Squamous cell carcinoma
	Tonsil	Side of the tongue	Squamous cell carcinoma
	Tonsil (left)	Tonsil (right)	unknown
Negative	Oropharynx	Thyroid gland	Papillary thyroid carcinoma
	Oropharynx	Side of the tongue	Squamous cell carcinoma, p16-negative
	Oropharynx	Lung	adenocarcinoma
	Tonsil	Base of tongue	Squamous cell carcinoma, p16-negative
	Tonsil (right)	Vallecula (left)	Squamous cell carcinoma
	Oropharynx	Soft palate	High grade dysplasia
	Base of tongue (right)	Larynx (left)	Squamous cell carcinoma
	Tonsil (left)	Hypopharynx (right)	Squamous cell carcinoma
	Tonsil	Lung	Squamous cell carcinoma
	Tonsil	Hypopharynx	Squamous cell carcinoma
	Tonsil (right)	Tonsil (left)	Squamous cell carcinoma (p16-positive)
	Tonsil	Hypopharynx	Squamous cell carcinoma
	Tonsil	Esophagus	Squamous cell carcinomas/adeno

**Table 8:** Multivariate Cox regression for SPT development in the head and neck area. Table 8 describes the results of the multivariate Cox regression of all SPT with respect to localization in the head and neck area (0 = SPT outside head and neck, 1 = SPT in head and neck) regressed on the independent variables tumor HPV positivity (negative = 0, positive = 1), smoking status (0 = <10 py, 1 = ≥10 py), alcohol status (0 = no alcohol abuse, 1 = alcohol abuse), and age (in years). * indicates statistical significance at the 5% significance level. Abbreviations: CI = confidence interval, HR = hazard ratio, py = pack years.

Multivariate Cox Regression for SPT Development in the Head and Neck	SPT in Head and Neck	
Parameter	HR (95% CI)	*p*-Value
Tumor HPV-positivity	1.102 (0.629–1.930)	0.734
Smoking (>10 py)	3.513 (1.154–10.693)	0.027 ^*^
Alcohol abuse	1.301 (0.730–2.319)	0.372
Age	0.934 (0.886–0.985)	0.012 ^*^
Number of observations	21	

## Data Availability

The data presented in this study are available on request from the corresponding author. The data are not publicly available due to ethical reasons.
